# Supplementary material for: Stacking Model‐Based Classifiers for Dealing With Multiple Sets of Noisy Labels
Source: Biom J. 2025 Mar 12;67(2):e70042. doi: 10.1002/bimj.70042 (PMC11898607; doi:10.1002/bimj.70042)
Supplement: Supplementary file 1 — Supporting Information [file BIMJ-67-e70042-s001.zip › density-based-ensemble-model-main/competitors_real_data/netlab-master/netlab-master/help/ppca.htm]

Netlab Reference Manual ppca


# ppca

## Purpose

Probabilistic Principal Components Analysis

## Synopsis

```
[var, U, lambda] = pca(x, ppca_dim)
```

## Description

`[var, U, lambda] = ppca(x, ppca_dim)` computes the principal component
subspace `U` of dimension `ppca_dim` using a centred
covariance matrix `x`. The variable `var` contains
the off-subspace variance (which is assumed to be spherical), while the
vector `lambda` contains the variances of each of the principal
components. This is computed using the eigenvalue and eigenvector
decomposition of `x`.

## See Also

`eigdec`, `pca`

---

**Pages:**
Index


---

Copyright (c) Ian T Nabney (1996-9)
